# Dictionnaire Universel de Matière Médicale, et de Thérapeutique générale, &c.

**Published:** 1833-10

**Authors:** 


*Dictionnaire Universel de Matière Médicale, et de Thérapeutique générale, &c.* Par F. V. Mérat et A. J. De Lens. À Paris, 1829—1833. Tom. I—V. 8vo. pp. 3422.

Five volumes of this Dictionary of the Materia Medica have already appeared, and one more will complete the work. The book certainly does credit to the industry and judgment of the compilers; it is remarkably rich in synonymes, as the following extract may show:

"Cétique (acide). V. *Acide margarique,* I. 38.

Cetracca, Cbetrach,* Citracca. Noms arabes du Cetrach officinarum, DC.

Cetraria Islandica. Ach., lichen d'Islande, *Lichen Islandicus,* L. Voy. *Lichen.*

Cetrivolo. Nom italien du concombre, *Cucumis sativus,* L.

Cetros. Nom grec du sain bois, *Daphne Gnidium,* L.

Cetus. Un des noms Latins du *Physeter macrocephalus,* Shaw.

Cevada. Nom portugais de l'*Hordeum vulgare,* L.

Cévadille. Nom du fruit du *Veratrum Sabadilla,* Retz.

Cévadique (acide). V. *Acide cévadique,* I. 32.

Ceynas. Un des noms indiens du *Bombax Ceiba,* L. (I. 637.)

Ceyx, Ceyeus. Noms donnés par Pline à l'*Hirundo esculenta,* L.

Cezé, Cezes, Cezerous. Noms languedociens du pois chiche, *Cicer arietinum,* L." Tom. ii. p. 193.

The reader must not suppose, however, that the bulk of the work consists of articles treated in this laconic style; *Opium,* for example, occupies eighteen pages.

We can fairly recommend this book to be purchased by medical clubs or societies. It will occasionally furnish scraps for our Collectanea.

